# Correction: Individuals with *JAK1* variants are affected by syndromic features encompassing autoimmunity, atopy, colitis, and dermatitis

**DOI:** 10.1084/jem.2023238704302024c

**Published:** 2024-05-06

**Authors:** Michael E. Horesh, Marta Martin-Fernandez, Conor Gruber, Sofija Buta, Tom Le Voyer, Eve Puzenat, Harry Lesmana, Yiming Wu, Ashley Richardson, David Stein, Stephanie Hodeib, Mariam Youssef, Jacob A. Kurowski, Elizabeth Feuille, Luis A. Pedroza, Ramsay L. Fuleihan, Alexandria Haseley, Alain Hovnanian, Pierre Quartier, Jérémie Rosain, Georgina Davis, Daniel Mullan, O’Jay Stewart, Roosheel Patel, Angelica E. Lee, Rebecca Rubinstein, Leyla Ewald, Nikhil Maheshwari, Virginia Rahming, Ivan K. Chinn, James R. Lupski, Jordan S. Orange, Vanessa Sancho-Shimizu, Jean-Laurent Casanova, Noura S. Abul-Husn, Yuval Itan, Joshua D. Milner, Jacinta Bustamante, Dusan Bogunovic

Vol. 221, No. 6 | https://doi.org/10.1084/jem.20232387 | April 2, 2024

The authors regret that Fig. 2, Fig. 3, and Table 2 contained errors in the originally published article. In Fig. 2 (g-j), an open square in the graph key identifying “fLUC” samples has been removed because these panels contain no data representing fLUC samples. In the Fig. 3 (a-f) bar graph key, the open square was erroneously labeled; this has been corrected to “fLUC,” and the label “WT” has been added to identify the black bars. In Table 2, “T” has been changed to “Y” in line 9 of the “Ultra-rare or rare Predicted GoF” row in the “(CADD)” column. The corrected figures and table are shown here, with the table correction indicated in red text. These corrections do not change the original conclusions of the article, and the figure legends remain unchanged. The errors appear in PDFs downloaded before April 30, 2024.

**Figure 2:**
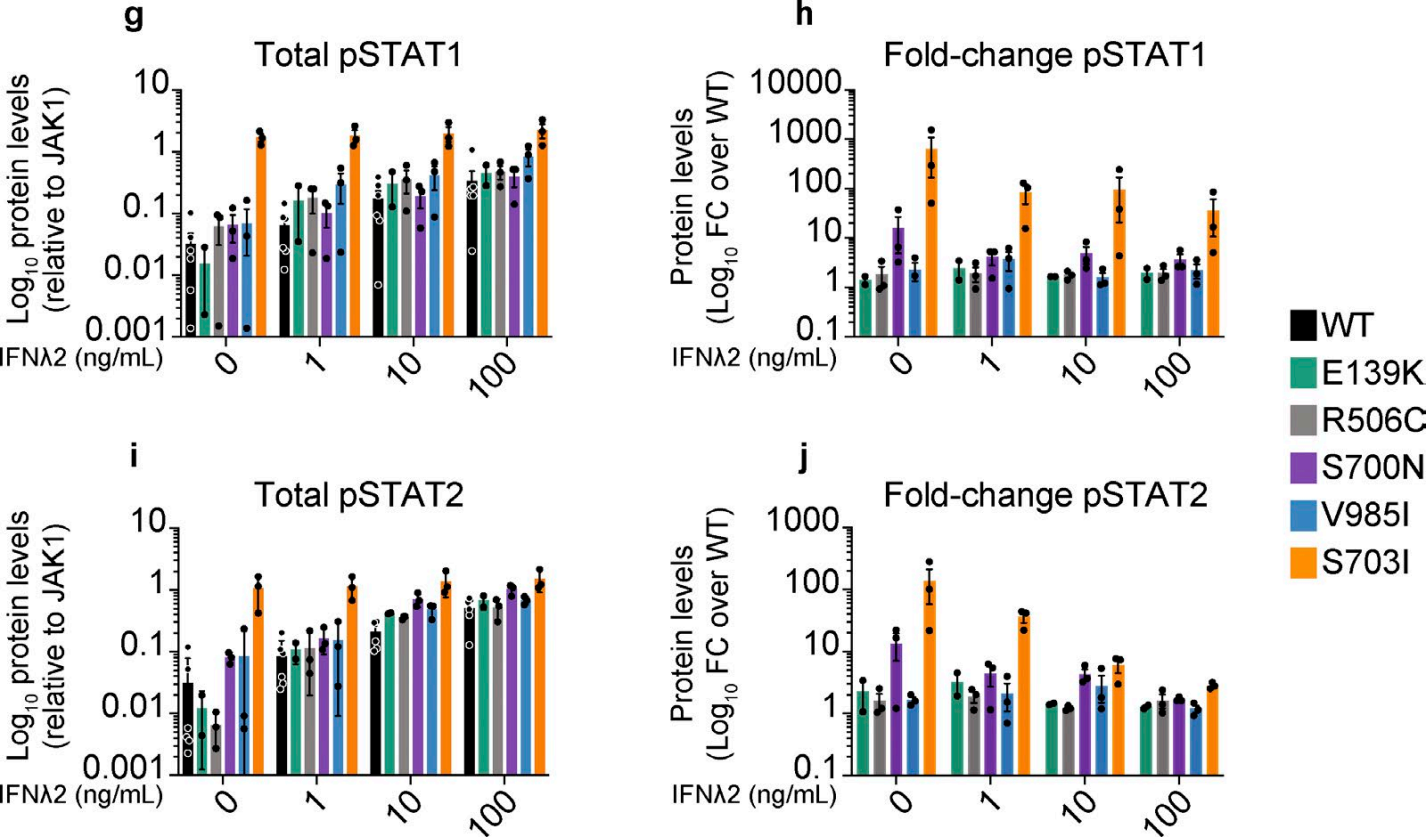


**Figure 3:**
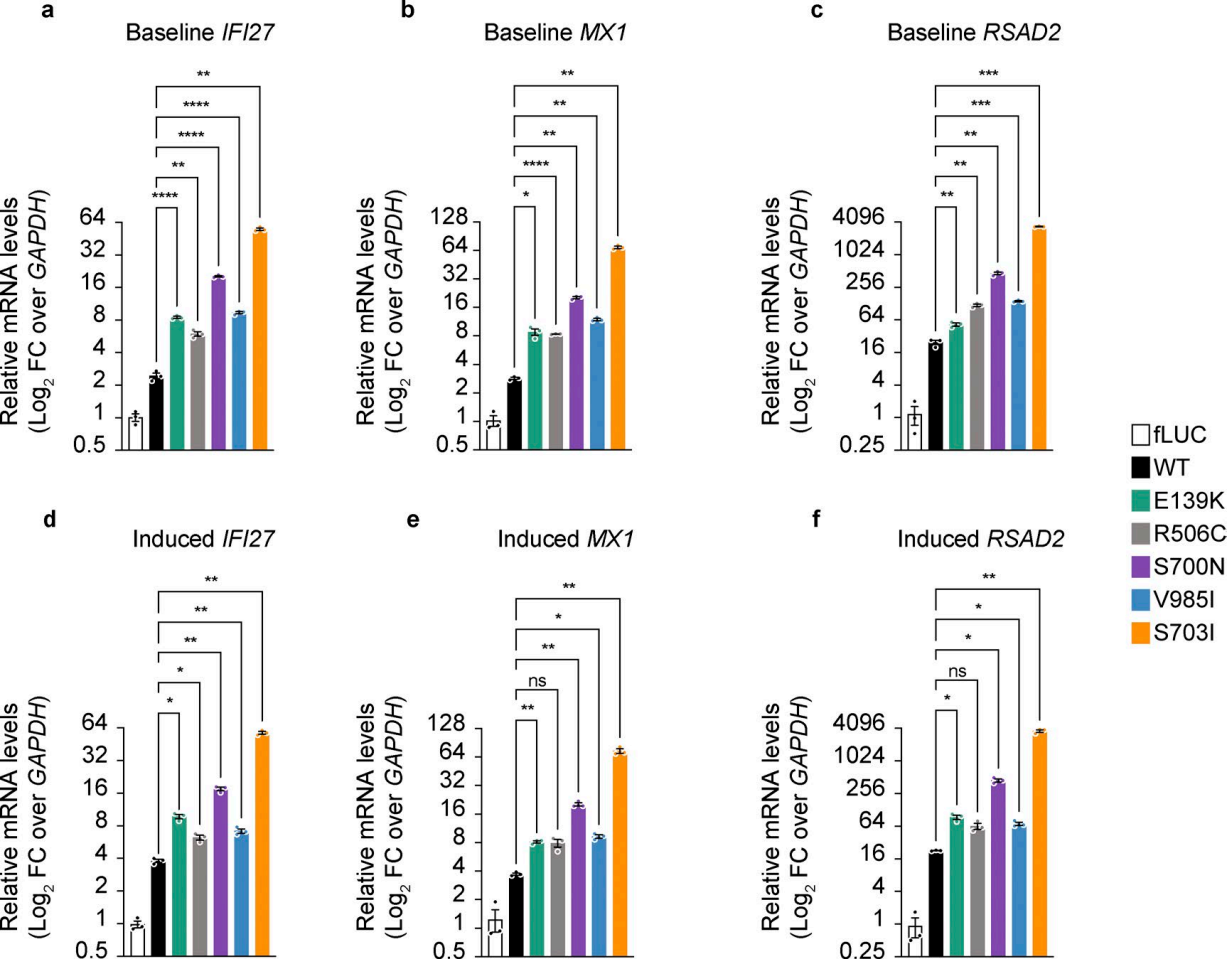


**Table 2.**
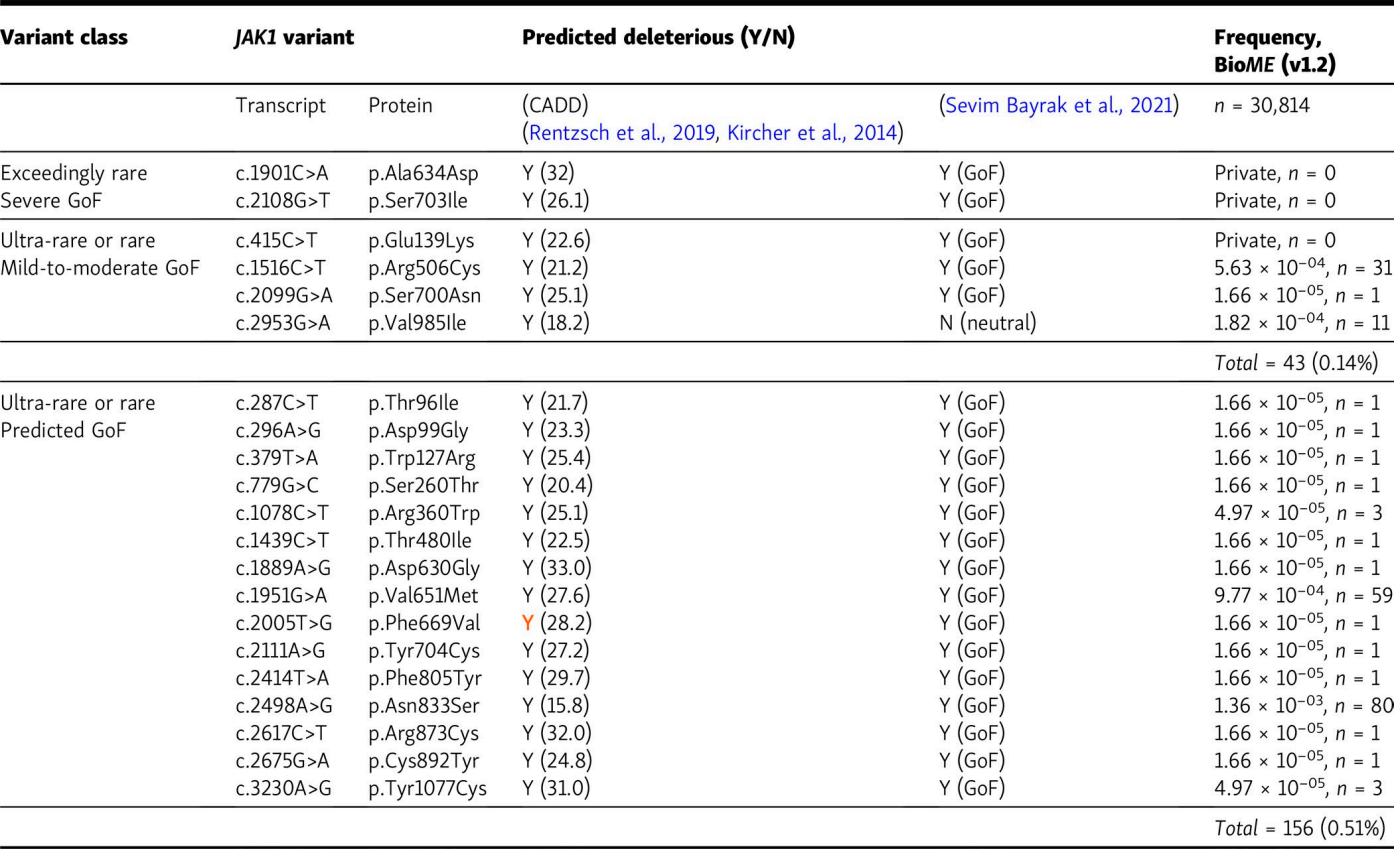
In silico prediction of GoF activity in *JAK1* variants

